# Phenotypic dynamics of microglial and monocyte-derived cells in glioblastoma-bearing mice

**DOI:** 10.1038/srep26381

**Published:** 2016-05-19

**Authors:** Clément Ricard, Aurélie Tchoghandjian, Hervé Luche, Pierre Grenot, Dominique Figarella-Branger, Geneviève Rougon, Marie Malissen, Franck Debarbieux

**Affiliations:** 1 Institut des Neurosciences de la Timone, Marseille, Aix-Marseille Université and CNRS UMR7289, France; 2 Services d’Anatomie Pathologique-Neuropathologique et de Pharmacie, Assistance Publique – Hopitaux de Marseille, Marseille, France; 3 Centre Européen de Recherche en Imagerie Médicale, Aix-Marseille Université, Marseille, France; 4 Centre de Recherche en Oncobiologie et Oncopharmacologie, INSERM UMR911 and Aix-Marseille Université, Marseille, France; 5 Centre d’Immunophénomique, Aix-Marseille Université UM2, INSERM, US012, CNRS UMS3367, Marseille, France; 6 Centre d’Immunologie de Marseille-Luminy, Aix Marseille Université UM2, INSERM, U1104, CNRS UMR7280, Marseille, France

## Abstract

Inflammatory cells, an integral component of tumor evolution, are present in Glioblastomas multiforme (GBM). To address the cellular basis and dynamics of the inflammatory microenvironment in GBM, we established an orthotopic syngenic model by grafting GL261-DsRed cells in immunocompetent transgenic LysM-EGFP//CD11c-EYFP reporter mice. We combined dynamic spectral two-photon imaging with multiparametric cytometry and multicolor immunostaining to characterize spatio-temporal distribution, morphology and activity of microglia and blood-derived infiltrating myeloid cells in live mice. Early stages of tumor development were dominated by microglial EYFP^+^ cells invading the tumor, followed by massive recruitment of circulating LysM-EGFP^+^ cells. Fluorescent invading cells were conventional XCR1^+^ and monocyte-derived dendritic cells distributed in subpopulations of different maturation stages, located in different areas relative to the tumor core. The lethal stage of the disease was characterized by the progressive accumulation of EGFP^+^/EYFP^+^ monocyte-derived dendritic cells. This local phenotypic regulation of monocyte subtypes marked a transition in the immune response.

Glioblastomas (GBM), the most aggressive form of glioma, are highly vascularized infiltrating brain tumors. They are resistant to current therapies and invariably fatal^1^. Strategies aiming at targeting GBM cells have so far proved largely unsuccessful. Increasing evidence shows that tumors progress by interacting with host cell types that reside in or are recruited to the tumor microenvironment^2^,^3^. GBM are notoriously immune-suppressive and GBM cells likely manipulate immune cells to support tumor expansion (for review^4^).

Inflammatory myeloid and lymphoid cells make up a substantial part of the GBM tumor mass; by some estimates as high as one third of the cells^5^. Cells with macrophage activity, usually called tumor-associated macrophages, frequently accumulate in the tumor stroma^4^. They are proposed to play an important role in suppressing immune responses to cancer cells, but their origin and heterogeneity have not been fully documented. Within the central nervous system (CNS) inflammatory reactions differ substantially from those of other tissues in several ways^6^,^7^. First, the CNS has its own resident innate immune cells composed of microglial cells^8^,^9^, recently shown to have a potential for self-renewal without the contribution of peripheral myeloid cells^10^. Second, the CNS parenchyma lacks conventional resident dendritic cells (DCs). Perivascular macrophages, vascular pericytes and subpopulations of microglia subsume the functions of mature DCs^11^,^12^,^13^. Third, the permeability of microvessels for extravasation of large molecules and blood cells is reduced by the blood–brain barrier (BBB) in the CNS in comparison to the rest of the body. Hence, CNS innate immune cells do not recruit the machinery of the adaptive immune response as efficiently as do conventional DCs in peripheral tissues. It is therefore crucial to obtain a more detailed understanding of the cascade and kinetics of immune responses inside the tumor bearing CNS as targeting of tumor-associated host cell subpopulations may represent a therapy to be explored. The phenotypic classification of GBM-associated immune cells is challenging. Attributing functions in GBM progression specifically to microglial cells^14^ or monocyte-derived cells, is a matter of debate due to the lack of specific markers for each subpopulation and their plastic phenotypes. Significant confusion in the current literature of myeloid subpopulations seems to result from inappropriate grouping of cells or from lack of a common method for distinguishing the various subpopulations. However, recent additions to the repertoire of antibody markers of DCs versus macrophages versus monocytes provided significant clarity to this situation^15^. Moreover, no research techniques have for example permitted unambiguous distinction between microglia and infiltrated monocytes in CNS tissue. Indeed, irradiation chimerism^16^ or transient parabiosis between the circulatory systems of a green fluorescent protein, GFP, labeled animal and of a wild type littermate^17^,^18^ are two heavy methodologies suffering from intrinsic limitations^19^. Recent breakthroughs in intravital cellular imaging have opened the way to longitudinal and functional characterization of the immune cell populations in various pathological contexts^20^.

We devised a clinically relevant syngenic GL261 GBM model in C57BL/6 mice that showed pronounced evolution of immune profile during tumor development. In order to gain insight in the respective involvement of resident microglia and circulating leucocytes across the different stages of tumor development, this model was implemented using multicolor fluorescent reporter mice. We analyzed the immune response with three complementary approaches: intravital spectral two-photon microscopy, multi-parametric cytometry and immunohistochemistry. Their combination enabled us to link information on cell dynamics, distribution, density and morphology to information on phenotypes and frequencies of immune cell populations during GBM progression.

## Results

### The LysM-EGFP//CD11c-EYFP mouse model permits the study of the dynamics of innate immunity during GBM progression

We developed a syngenic orthotopic GBM model in immunocompetent C57BL/6 mice. This model recapitulates the typical features of the tumor and is suitable for intravital two-photon imaging and multiparametric flow cytometry analyses. We crossed LysM-EGFP^21^ and CD11c-EYFP^22^ mice to obtain LysM-EGFP//CD11c-EYFP double-transgenic animals to facilitate visualization of peripheral myeloid cells, microglia and DCs, respectively. Among the described murine models to study GBM initiation and development^23^, we selected a reproducible graft model in which the tumor develops in the brain at a depth accessible to *in vivo* two-photon imaging. This model employs the stereotaxic injection of a spheroid of fluorescent GL261 glioma cells stably expressing DsRed into the superficial layers of the parietal cortex (approximately 250 μm deep) of LysM-EGFP//CD11c-EYFP and occasionally of Thy1-CFP//LysM-EGFP//CD11c-EYFP mice to highlight subsets of cortical pyramidal neurons. The dura-mater is sealed with an agarose hemi-bead and the bone above the injection site is replaced by a glass window (4–5 mm in diameter). The cranial window allows a non-invasive follow-up of the events throughout the disease progression^24^.

We evaluated the effect of our grafting protocol on cortical architecture and inflammation by performing the whole surgical protocol except the injection of the GL261-DsRed glioma spheroid (sham mice). We showed by two-photon imaging of the Thy1-CFP//LysM-EGFP sham mice, that the adjacent neuronal cyto-architecture was not affected by the surgery (Suppl. Fig. 1). Imaging of the LysM-EGFP and CD11c-EYFP sham mice from day 15 (D15) to D33–34 post-surgery revealed that LysM-EGFP^+^ cells were virtually absent from the brain parenchyma whereas rare CD11c-EYFP^+^ cells could be seen. A few LysM-EGFP^+^ and CD11c-EYFP^+^ cells were present as a monolayer at the surface of the sealing agarose hemi-bead (Suppl. Fig. 1). Their density did not vary over time and the CD11c-EYFP^+^ cells in the parenchyma did not move towards the hemi-bead during the 20-day observation period.

**Figure 1:**
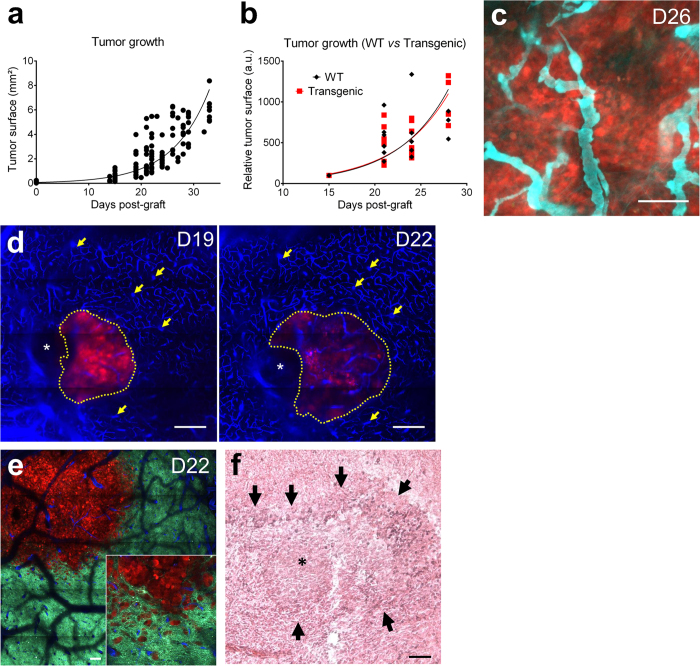
A syngenic glioblastoma mouse model allows intravital two-photon imaging of disease progression. **(a)** DsRed^+^ Glioma261 growth curve measured by fluorescence macroscopy (n = 40 mice). **(b)** Tumor growth curves in WT (black, diamonds, n = 9 mice) and transgenic (red, squares, n = 13 mice) animals. **(c)** Intravital two-photon imaging of GBM cells and tumor vasculature at day 26 (D26) post-graft. Red: tumor cells; cyan: vasculature. Scale-bar: 100 μm. **(d)** Intravital two-photon imaging of GBM cells and brain vasculature on the same animal at D19 and D22 post-graft. Red: tumor cells; blue: vasculature; asterisk: Sephadex hemi-bead; arrows: stable vascular landmarks, dotted line: tumor margin. Scale-bar: 100 μm. **(e)** Intravital two-photon imaging of GBM and surrounding brain parenchyma at D22 post-graft. Note the escape of the tumor cells that invade the brain parenchyma at tumor margin. Red: tumor cells; blue: vasculature; cyan: neurons. Inset: zoom on escaping cells. Scale-bar: 100 μm. **(f)** Haematoxylin-Eosin staining of a GL261 tumor. Asterisk: necrotic area; arrows: peri-necrotic pseudopalissades. Scale-bar: 50 μm.

We then implanted GL261-DsRed glioma spheroids in both wild type and LysM-EGFP//CD11c-EYFP transgenic mice. Tumor growth was measured by fluorescence macroscopy every 3 to 4 days from D15 until D33, the final stage of the disease at which all mice lost weight and some died. Tumor expansion (n = 40) fits with an exponential curve (R^2^ = 0.8378) (Fig. 1a) with slow growth until D15 followed by rapid expansion afterwards. No significant difference in tumor growth was seen between double transgenic (n = 13, Tau = 0.18; R^2^ = 0.8619) and wild-type (n = 9, Tau = 0.17 R^2^ = 0.783) mice (Fig. 1b), indicating that the introduction of fluorescent tags did not modify tumor development. Interestingly, although on average tumor surface increased exponentially over 4 weeks, the daily progression rate significantly decreased from 1.2 fold at D21 to 1.09 at D28 (n =  17, *p* < 0.001).

Recurrent two-photon imaging of GL261-DsRed grafted mice receiving an injection of Cascade blue to highlight blood vessels revealed that the tumor elicited vascular remodeling in its core (Fig. 1c,d), while individual tumor cells invading the adjacent healthy brain parenchyma can be observed (Fig. 1e). Histo-pathological examinations also revealed a necrotic core in the neoplasm (Fig. 1f).

Altogether these data showed that our syngenic orthotopic model in LysM-EGFP//CD11c-EYFP mice mimics GBM physiopathology (angiogenesis, invasiveness of parenchyma, necrotic core) and permits investigation of the changes in immunological parameters during GBM development by recurrent *in vivo* two- photon spectral imaging.

### Imaging LysM-EGFP and CD11c-EYFP cells accumulation dynamics and distribution during GBM progression

**Figure 2:**
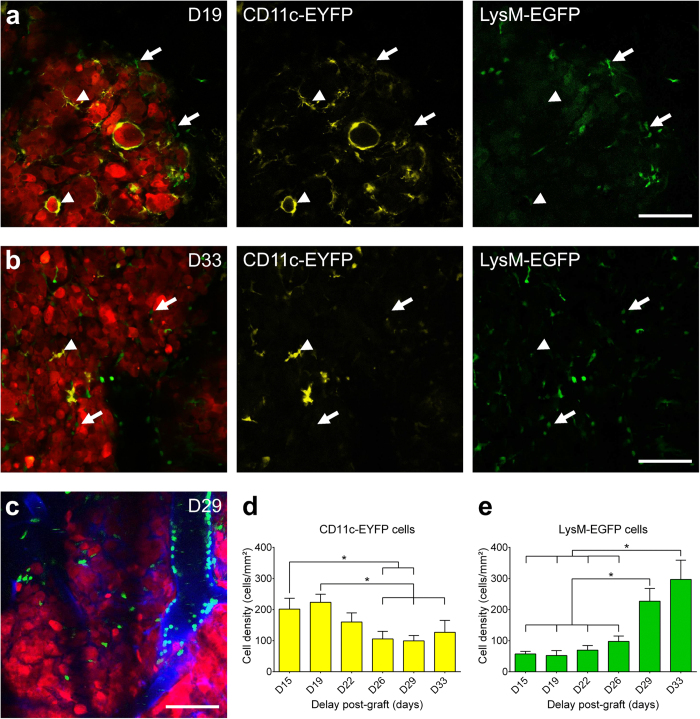
Intravital two-photon imaging of CD11c-EYFP and LysM-EGFP cells dynamics during GBM progression. **(a**,**b)** Intravital two-photon imaging of tumor (red), CD11c-EYFP (yellow, arrowheads) and LysM-EGFP (green, arrows) cells at **(a)** early stage (D19) and **(b)** terminal stage (D33) of GBM progression. Scale-bar: 100 μm. **(c)** Amoeboid LysM-EGFP cells (green) accumulation in and around blood vessels (blue) at late stage (D29) of GBM progression. Scale-bar: 100 μm. **(d**,**e)** Evolution of CD11c-EYFP (**d**) and LysM-EGFP (**e**) cells densities inside the tumor during GBM progression (n = 12 to 6 mice depending on the timepoint). *p < 0.05.

To obtain information on temporal fidelity and statistical significance using a minimal number of animals, we repeatedly imaged the same LysM-EGFP//CD11c-EYFP mice every 3 to 4 days from D15 to D33 post-grafting (n = 12 to 6, depending on the time-point) (Fig. 2). Whatever the time post-graft, CD11c-EYFP^+^ cells were never observed in the circulation. At D19 of GBM progression, CD11c-EYFP^+^ cells were found to accumulate intermingled with tumor cells inside the tumor (224 ± 26 cells/mm^2^) (Fig. 2a,d). They were scarce in the parenchyma surrounding the tumor. We observed CD11c-EYFP^+^ cells wrapped around GBM cells (Fig. 2a). By contrast, LysM-EGFP^+^ cells, absent from healthy tissue, were observed in the circulation and tumor blood vessels suggesting that they were recruited from the periphery to the tumor site. In the pathological brain, LysM-EGFP^+^ cell density was low (70 ± 15 cells/mm^2^) until D22, with approximately two third of the cells observed at the periphery of the tumor in a non-clustered manner (Fig. 2a,e).

At the latest stages (>D26) of GBM progression, CD11c-EYFP^+^ cells were still present, although their density decreased by half inside the tumor (D19, 224 ± 26 cells/mm^2^; D29, 100 ± 17 cells/mm^2^; Fig. 2b,d). On the other hand, the density of LysM-EGFP^+^ cells increased strongly during GBM progression, resulting in a 4-fold increase in density between D22 and D33 (D22, 70 ± 15 cells/mm^2^; D33, 297 ± 62 cells/mm^2^) (Fig. 2e). LysM-EGFP^+^ cells invaded the tumor core (Fig. 2b), most likely due to extravasation from localized intraluminal accumulations of cells (Fig. 2c). These changes in the relative densities of fluorescent cells were rather sharp and preceded the lethal phase of the disease. This dynamic recruitment was triggered by the tumor development, as it was not observed in sham-operated LysM-EGFP//CD11c-EYFP mice. In the tumor, the CD11c-EYFP^+^ and the LysM-EGFP^+^ cells seemed to have reciprocal dynamics, as the highest densities of CD11c-EYFP^+^ cells corresponded to the lowest densities of LysM-EGFP^+^ cells and vice-versa.

### Phenotyping innate immune responses during GBM progression

To document the nature of the immune response and the degree of heterogeneity found in the LysM-EGFP^+^ and CD11c-EYFP^+^ populations during tumor progression, we performed multi-parametric flow cytometry analyses. We collected and dissociated tumors and neighboring cortex tissues of PBS perfused individual mice taken before (D21) and after (D28) tumor invasion by LysM-EGFP^+^ cells. Sham-operated mice were analyzed in parallel to tumor-implanted animals.

**Figure 3:**
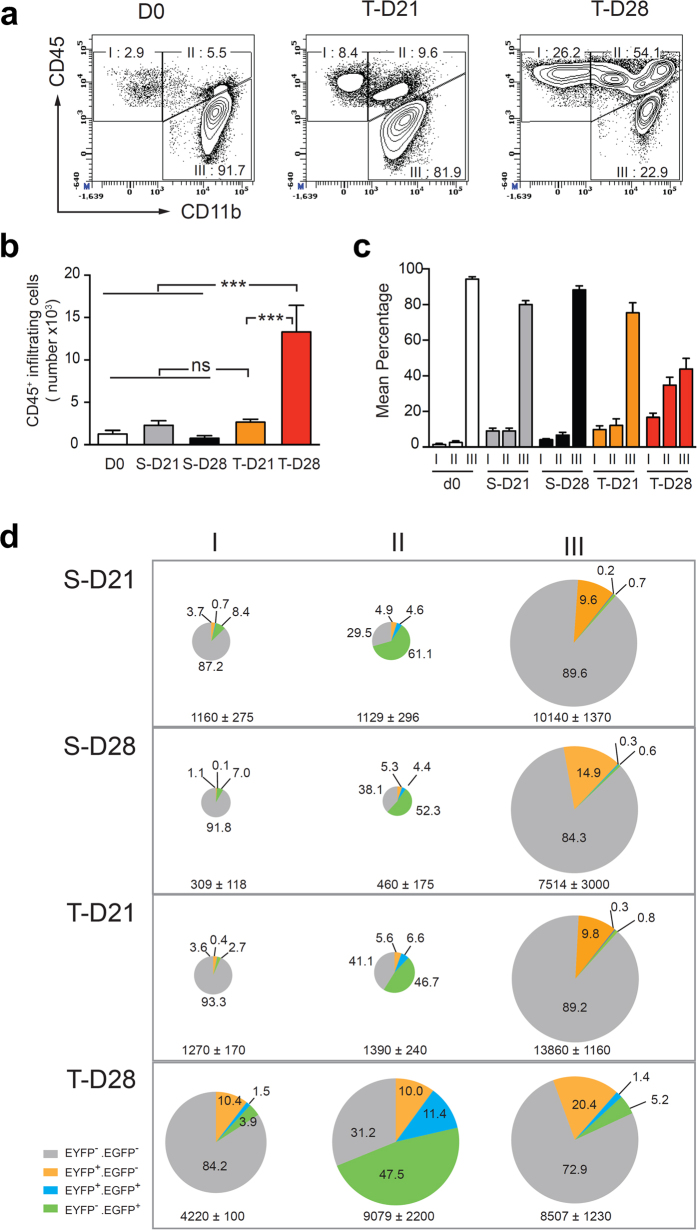
Flow cytometric analysis of brain infiltrating leukocytes during tumor progression. **(a).** CD45^+/low^ leukocytes extracted from the brain before (D0), or 21 (T-D21) or 28 (T-D28) days after tumor grafting were analyzed for the expression of CD11b and CD45 and subdivided into CD45^+^CD11b^−^ (denoted as I), CD45^+^CD11b^+^ (denoted as II), and CD^45 low^CD11b^+^ (denoted as III) populations. The percentage of cells in each population is indicated. **(b).** Histogram depicting the number of brain infiltrating cells corresponding to pooled populations I and II (see a) in control (D0), sham-operated (S-D21 and S-D28) or and tumor implanted (T-D21 and T-D28) mice. Pooled data from individual mice are represented as mean ± SEM (D0: n = 5, S-D21: n = 7, S-D28: n = 5, T-D21: n = 12 and T-D28: n = 13). Statistical significance was evaluated by a Mann Whitney test: ***p < 0.001, ns: non significant. **(c)** Relative representation of populations I, II and III (see a) in brain in the different groups of mice. Data are presented as the mean ± SEM in each group. (**d**) Percentages of LysM-EGFP^+^ (green), CD11c-EYFP^+^ (orange) and LysM-EGFP^+^/CD11c-EYFP^+^ (blue) cells among populations I, II and III (see a) found in the brain of sham-operated (S-D21 and S-D28) or tumor implanted (T-D21 and T-D28) mice. The surface of the pie charts is proportional to the absolute cell number in the specified population. Cell numbers are indicated under each pie chart as mean ± SEM (S-D21: n = 4, S-D28: n = 4, T-D21: n = 6 and T-D28: n = 6).

We validated a 19-parameter flow cytometry panel (Suppl. Table 1, Suppl. Figs 2 and 3) compatible with the simultaneous detection of the two EGFP and EYFP fluorescent reporter proteins. Analysis of living cells with the surface markers CD45 and CD11b allowed discrimination of three main populations, denoted as populations I (CD45^high^/CD11b^−^), II (CD45^high^/CD11b^+^) and III (CD45^low^/CD11b^+^, corresponding to microglial cells^25^) (Fig. 3a). In sham-operated mice (Fig. 3c), the explanted tissue was dominated by microglial cells (>90% of analyzed cells), whereas populations I and II accounted for <10% of the analyzed cells. At D21, the brain immune profile of tumor bearing animals, determined by flow cytometry was essentially the same as for control and sham-operated animals, both in term of cells numbers and fluorescent protein expression (Fig. 3d). The late stage of tumor development was characterized by a dramatic change of immune profile. Indeed, at D28, the representation of population III decreased in favor of populations I and II, concomitantly with the five-fold increase in the number of CD45^+^ infiltrating cells (I + II) when compared to tumor bearing mice at D21 (Fig. 3a–c).

Approximately 95% (90.9% S-D21, 97.6% S-D28) of the CD11c-EYFP^+^ cells in sham-operated mice belonged to population III. They were therefore identified as a subpopulation of resident microglia representing roughly 12% (9.6% S-D21, 14.9% S-D28) of the total microglial population (Fig. 3d). At D21, more than 90%, (91.2 ± 1.3%) of EYFP^+^ cells co-expressed Iba1, a marker for microglia in the healthy CNS (Suppl. Figs 4 and 5c). On the other hand, 80% of the LysM-EGFP^+^ cells (80.4% S-D21, 78.3% S-D28) belonged to population II where they accounted for roughly 50% of the cells. They were 10-fold less numerous in population I, and accounted for less than 1% of population III (Fig. 3d).

The massive infiltration of peripheral immune cells at late tumor stages was accompanied by a diversification of phenotypes of the fluorescent cells as indicated by the detection of CD11c-EYFP^+^ cells in populations I (10.4%), and II (10%). We also observed the emergence of double-labeled cells essentially in population II (11.4%) (Suppl. Fig. 2) despite the absence of such cells in the pool of circulating cells.

Altogether these data confirmed that the resident microglia (population III) is the major population of immune cells involved in the early phase of the tumor growth and that the number of blood-derived infiltrating cells remains low. They also highlighted the delayed infiltration of cell populations I and II during the fourth week of tumor growth, in agreement with the invasion of the tumor core by LysM-EGFP^+^ cells (population II) that was observed by two-photon imaging from D26 onward (Fig. 2e). This correlation of terminal progression of the tumor with expansion of populations I and II raised issues about their cell composition and function.

**Figure 4:**
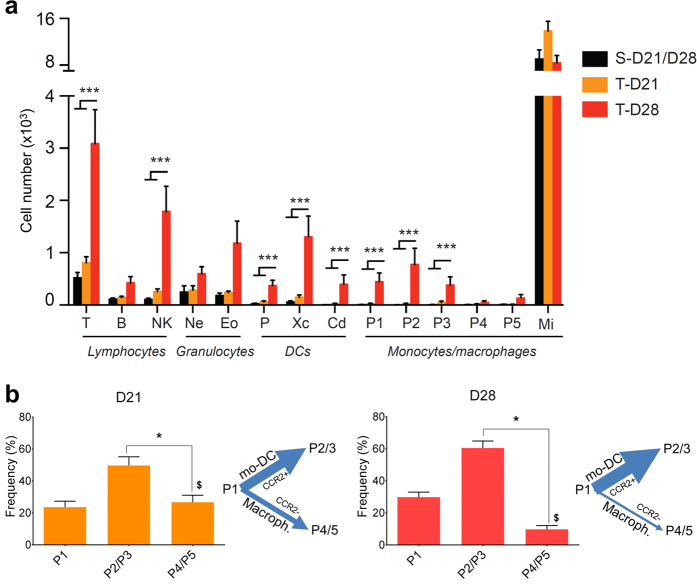
Multiparametric analysis of brain infiltrating cells allowed identification of all infiltrating leukocytes. **(a**) Absolute numbers of lymphocytes (T: T cell, B: B cell and NK: NK cell), granulocytes (Ne: Neutrophils and Eo: eosinophils), DCs (P: pDC, Xc:XCR1^+^DC and Cd: CD11b^+^DC) and monocytes/macrophages (Mi: microglia and P1, P2, P3, P4, P5 cells as defined in^15^ in the brain of sham-operated (S-D21 and S-D28 are grouped) and tumor-implanted (T-D21; T-D28) mice at D21 and D28. Data are presented as mean ± SEM (n = 12). Multiple comparisons were made using one-way ANOVA. Statistically significant differences are observed between T-D28 and S-D21/D28 or T-D21 with a ***p < 0.001 for most of the populations with the exception of B cells, neutrophils, eosinophils, P4, P5 and microglia. No statistically significant differences are observed between S-D21/28 and T-D21. (**b**) Frequency distribution and differentiation chart of the precursor P1 population, the mo-DC population (P2–P3) and the Macroph. population (P4-P5) at D21 and D28. mo-DC: monocyte-derived DCs; Macroph.: Macrophages. *p < 0.01 P2/3 *vs*. P4/5; ^$^*p* < 0.01 D21 *vs*. D28, Mann-Whitney test.

To this end, successive gating on 13 membrane proteins in addition to CD45 and CD11b (Suppl. Figs 3 and 4, Suppl. Table 1), allowed us to untangle and quantify the corresponding populations of leucocytes (Fig. 4a). Comparing sham-operated and tumor-bearing mice at D28, we observed significant increases in T lymphocytes (three-fold increase), natural-killer cells and in all DC subpopulations (XCR1^+^ DCs, CD11b^+^ DCs and plasmacytoid DCs, monocyte-derived DC P2 and P3 as well as in their precursor P1). Microglial cells outnumbered DCs by 45-fold until D21 but were only 2.7-fold more numerous at D28.

It is noteworthy that one third of the total DCs present in the brain during tumor progression were monocyte-derived DCs (24% at D21, 37% at D28), distributed in subpopulations of different maturation stages, whose size and fate was likely influenced by the tumor microenvironment. The precursor P1 subpopulation differentiated into a predominant CCR2^+^ population composed of P2 and P3 monocyte-derived DCs and into a smaller CCR2^−^ population composed of P4 and P5 macrophages. Differences between the densities of monocyte-derived DCs and macrophages were more pronounced during the latest stage of the disease (Fig. 4b).

### Uncovering the immunophenotype of transgenically labeled cells

**Figure 5:**
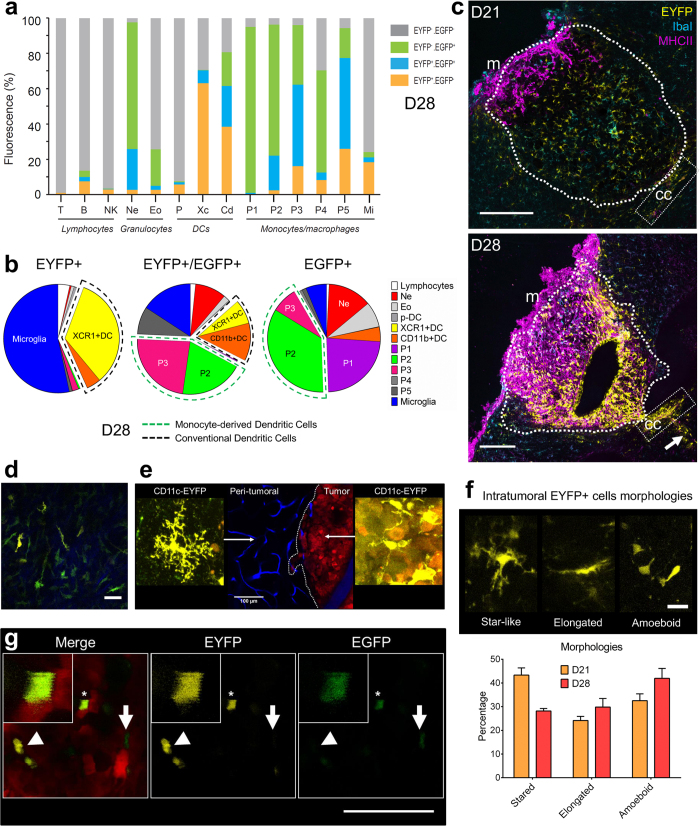
Immunophenotyping of LysM-EGFP and CD11c-EYFP cells. (**a**) Representative evaluation of the percentage of LysM-EGFP and CD11c-EYFP expressing fluorescent cells in each of the populations specified in Fig. 4a, in the brain of tumor bearing mice at D28. Values are expressed as cumulated percentages. (**b**) Distribution of the different immune populations in the whole EYFP^+^, EYFP^+^/EGFP^+^ and EGFP^+^ populations at D28. (**c**) Multicolor immunostaining of EYFP^+^ (yellow), Iba1^+^ (cyan) and MHCII^+^ (magenta) cells in sagittal sections of tumor bearing brains at D21 (top) and D28 (bottom). (m) meninges; (cc) corpus callosum. Dotted-lines highlight tumor margins. Dotted-squares: EYFP^+^ cells in the (cc). Arrow: Flow of EYFP^+^ cells arising for deep brain structures such as ventricles. Scale-bar: 200 μm. (**d**) Dura-mater specific morphologies as observed *in-vivo* by two-photon imaging of EYFP^+^ (yellow, arrow) and EGFP^+^ (green, arrowhead) cells above the tumor at D15 post-graft. Note the second-harmonic signal (blue) characteristic of the fibers of the meningeal layer. (**e**) Main morphologies of EYFP^+^ (yellow) cells imaged *in vivo* in peri-tumoral and tumor areas. Red: tumor cells; blue: vasculature. (**f** ) Distribution of the three main morphologies (star-like elongated and amoeboid) of EYFP^+^ cells observed *in vivo* by two photon imaging at D21 and D28. Scale bar: 20 μm. **(g)** *In vivo* two-photon image of a CD11c-EYFP^+^/LysM-EGFP^+^ cell taken at D26 post-graft (asterisk: CD11c-EYFP^+^/LysM-EGFP^+^ cell, arrowhead: CD11c-EYFP^+^ cell, arrow: LysM-EGFP^+^ cell). Scale-bar: 100 μm.

We used the expression profile of EYFP^+^ and EGFP^+^ cells determined by flow cytometry analysis among the different cell populations combined with immunostaining to describe their patterned distribution during tumor development (Fig. 5). At D28 multiparametric data indicated that in the brain of tumor-bearing mice EYFP expression was mainly encountered in a subset of the main microglial population (20%. of the whole microglial population, T-D28 in Fig. 3d), as well as in two-thirds of the XCR1^+^ DCs (60% of the whole XCR1^+^ DC population), and in nearly half of CD11b^+^ DCs (40% of the whole CD11b^+^ DC population) (Fig. 5a). Given the relative incidence of these cell populations, we inferred that the non-microglial EYFP^+^ cells we observed at D28 were mostly XCR1^+^ DCs since they outnumbered CD11b^+^ DCs by nearly eight-fold (Fig. 5b).

Moreover, quantitative analysis of multicolor immunostained saggital brain slices at D28 showed that EYFP^+^/MHCII^+^ DCs (mainly XCR1^+^ DCs) represented 31% of the intratumoral EYFP^+^ cells. They were densely accumulated in the superficial tumor layer (Fig. 5c). On the other hand, DCs exhibiting a lower level of staining for MHCII were grouped along the corpus callosum that invaded the deepest part of the cortical tumor (Fig. 5c,d). In the tumor core, 75% of EYFP^+^ cells observed were Iba1^+^, which supported their microglial identity. Their star-shape and the multiple cytoplasmic extensions observed by two-photon imaging also identified them as microglia (Fig. 5e). The cells we observed inside the tumor were less ramified than in the tumor periphery and became amoeboid during tumor development, as expected from a progressive evolution of their activation state (Fig. 5e,f). Immunostaining indicated that 20% of these cells expressed MHCII at D28, thus revealing their antigen presentation ability. It was noteworthy that MHCII expression was more frequent in the non-fluorescent microglial population since 45% of the non fluorescent Iba1^+^ population was MHCII^+^ at D28. By contrast at D21 MHCII was present in less than 2% of EYFP^+^ microglia and in 5% of the non labeled Iba1^+^ population (Fig. 5c). Therefore, despite the lack of selectivity of the transgenic EYFP labeling, our data indicate that EYFP^+^ cell subpopulations can nevertheless be identified in the living animal based on their patterned distributions relative to the tumor core.

Similarly, Ly6G^+^ immunostaining data indicated that at D21 more than 80% of the EGFP^+^ cells accumulating in the tumor were monocyte-derived cells (Suppl. Fig. 5) and not EGFP^+^ neutrophils despite the high number of neutrophils extracted from the tissue (Fig. 4a).

Two-photon microscopy and FACS data further suggested that the subpopulations of EGFP^+^ monocyte-derived cells (P1 to P5) could be differentiated by the graded co-expression of EYFP with EGFP (Fig. 5b,g). In agreement, double labeling was absent from the immature P1 population but found in P2 and P3 monocyte-derived DCs that represented altogether 43% of the double-labeled EGFP^+^/EYFP^+^ cells at the latest stage. The low representation of the P4 and P5 populations at D28 over the P2 and P3 population (Figs 4b and 5b) prevented the clear identification of these cell phenotypes in the double-labeled population during two-photon microscopy observations. The accumulation of these double-labeled cells inside the tumor could be considered as a biomarker of a transition in the immune response (Fig. 5b). Moreover, absence of these double-labeled cells in the blood stream strongly suggested that the up-regulation of EYFP expression was triggered by the tumor environment.

### Use of dynamic intravital imaging to gain insight into the function of LysM-EGFP^+^ and CD11c-EYFP^+^ cells

**Figure 6:**
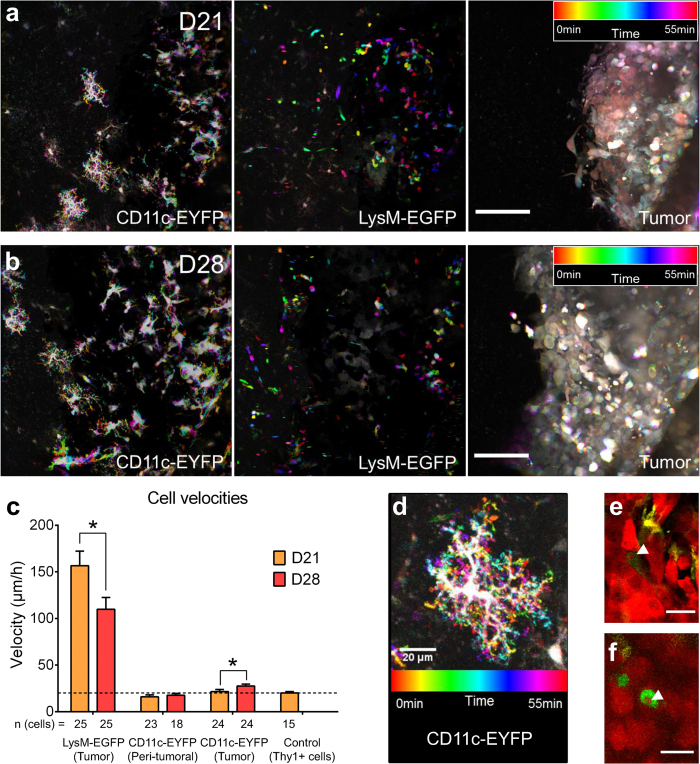
CD11c-EYFP and LysM-EGFP cells morphology and motility during GBM progression. (**a,b**) Time-color coded images of LysM-EGFP^+^, CD11c-EYFP^+^ and tumor cells taken at (**a**) D21 and (**b**) D28 post-graft over a 55-min observation period. Highly motile cells and structures appear in color, immobile cells and structures appear in white. Scale-bar: 100 μm. (**c**) CD11c-EYFP^+^ and LysM-EGFP^+^ cells velocities at D21 (orange, n = 5 mice) and D28 (red, n = 4 mice) post-graft. Thy1^+^ cells (neurons) were used to assess the lower sensitivity threshold of the measurements (dotted-line). *p < 0.05. (**d**) Time-color coded image of a CD11c-EYFP^+^ star-shaped cell taken in the peri-tumoral area at D21 over a 55 min observation period. Note the highly motile cytoplasmic protrusions (color) and the immobile cell body (white). Scale-bar: 20 μm. (**e**,**f**) Intravital two-photon imaging of a (**e**) CD11c-EYFP^+^ and (**f** ) LysM-EGFP^+^ cell that have phagocytized a DsRed expressing tumor cell fragment (red, arrowhead). Scale-bar: 20 μm.

To gain insight into the functional diversity of the fluorescent cell populations described by FACS and immunostaining, we performed intravital time-series imaging at D21 and D28, respectively. Acquisitions were performed from 0 to 200 μm below the dura-mater inside the tumor every 5 min for 1 to 3 hours. We took advantage of time-color coded projections to highlight the mobility of fluorescent cells. At D21 we observed that amoeboid EGFP^+^ cells were highly motile (157 ± 16 μm/h, n = 5 mice and 25 cells) and that they could move randomly inside the tumor (Fig. 6a,c, Suppl. Fig. 6, Suppl. Movie 1). By contrast, CD11c-EYFP^+^ somas were immobile over a one-hour observation period (n = 5 mice and 24 cells, Fig. 6a,c, Suppl. Movie 1) and only their fine processes were dynamically and quickly scanning the environment (Fig. 6d) as reported for microglia. At D28, we evidenced a modest but significant increase in the average movement of CD11c-EYFP somas in the tumor (Fig. 6b,c, Suppl. Movie 2), correlated with the increase in the number of XCR1 DCs. We also observed a 30% decrease in the velocity of LysM-EGFP^+^ cells (110 ± 13 μm/h, n = 24 p < 0.05), concomitant with the diversification of cell phenotypes.

These average migration speeds resulted from phases of acceleration and arrest that might correspond to interactions with other immune cells types (Suppl. Fig. 6, Suppl. Movies 1, 3). Indeed, such episodes were reminiscent of cellular interaction events described in the case of adaptive immune cells^26^. We occasionally (n = 3) also observed lasting interactions between EGFP^+^ monocyte-derived DCs and EYFP^+^ microglia at D28. Contacts between EGFP^+^ or EYFP^+^ cells and DsRed GL261 cells were frequent and in some instances resulted in phagocytic events as attested by the presence of red inclusions in the cytoplasm of some of these cells at D28 (Fig. 6e,f). Such red inclusions were rare in LysM-EGFP^+^ cells, but frequent in the population of CD11c-EYFP^+^ cells (30% of the cells n = 3 mice at D21 and D28, 5 ROI/mouse), as expected from the reported phagocytic competence of microglia^27^.

### Expression, distribution and morphology of LysM and CD11c in human GBM

**Figure 7:**
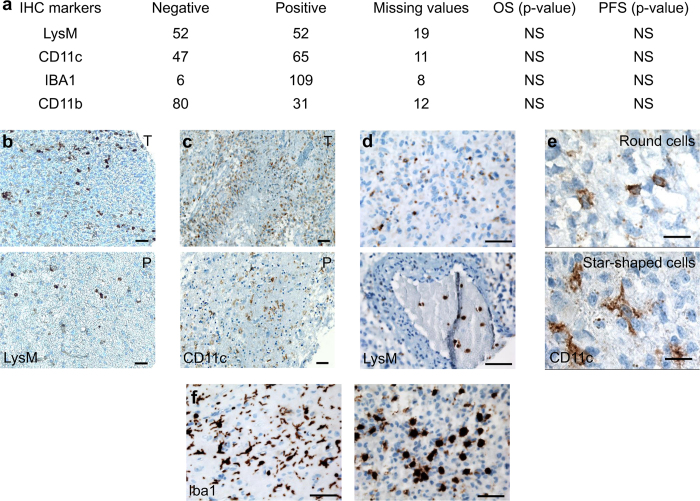
Immunohistochemistry of inflammation markers in a cohort of 123 patients with GBM tumors. (**a**) Number of negative and positive immuno-labeled samples for LysM, CD11c, Iba1 and CD11b inflammation markers. Overall survival (OS) and progression free survival (PFS) were calculated for negative and positive groups for each marker and were compared using the log-rank test (*p*-value). N.S.: not significant. (**b**) Representative LysM distribution in GBM tumor. LysM staining was denser in the core (T, top) than in the periphery of the tumor (P, bottom). (**c**) Representative CD11c distribution in GBM tumor. CD11c staining was denser in the core (T, top) than in the periphery of the tumor (P, bottom). (**d**) Representative picture of LysM labeling with positivity in the tumor core (top) and inside vessels (bottom). (**e**) CD11c^+^ cell morphologies: round (top); star-shaped (bottom) (**f** ) Representative pictures of Iba1^+^ cell morphologies: round (right) and star shaped (left). Scale-bars: 50 μm.

To expand the potential knowledge gained in mice into an ability to translate it to humans, we searched for the expression of LysM, CD11c, CD11b and Iba1 markers in human GBM biopsies. Indeed, data showed that it is possible to correlate the subsets of mononuclear phagocyte cells found in the mouse with those present in the human^28^. Immunohistochemical analysis of the expression of these markers was performed on a cohort of GBM tumors (n = 123). Despite a lack of significant prognostic value of these markers within our cohort, it was of interest that we found that 50% of human GBM expressed LysM and that 58% expressed CD11c (Fig. 7a). As observed by imaging (Fig. 2), we noticed a differential distribution of the immune cells between the core (defined as the most contrasted areas following gadolinium injection) and the periphery, with higher densities of LysM^+^ and of CD11c^+^ cells inside the tumor compared to the tumor margin (Fig. 7b,c). Among LysM positive GBM samples, 61.5% expressed LysM only in the tumor core, 27% expressed LysM both in the tumor core and its periphery, while 11.5% expressed LysM only inside the vessels (Fig. 7d). LysM was preferentially expressed in biopsies presenting a significant level of CD11b immunostaining (*p* = 0.044), which supported the existence of a double positive LysM^+^/CD11b^+^ population in human GBM as shown in mice. Twenty seven per cent of patients were positive both for LysM and CD11c. The highest CD11c densities corresponded to weak LysM densities as expected from the mutual exclusion of the LysM^+^ and CD11c^+^ populations observed in mice. CD11c^+^ cells had either ramified or round morphologies (Fig. 7e). Similar ramified and round morphologies were also observed among the Iba1^+^ cells (Fig. 7f) in 95% of human biopsies (Fig. 7a). Taken together these results showed that LysM and CD11c are expressed in the human GBM microenvironment and most likely reflect the presence of similar cell populations as those found in the mouse.

## Discussion

In this study we detailed the dynamics of the innate immune response associated with GBM progression in immunocompetent transgenic reporter mice by combining the most advanced cortical imaging methodology^29^ with high content flow cytometry analysis^15^. Our results demonstrate the complementarity of multiparametric cytometry to untangle the diversity of cell phenotypes involved in the innate immune response and microscopy to delineate the dynamics and compartmentalization of this immune response between intratumoral and peritumoral areas. For example at early stages, MHCII^+^ cells identified by cytometry were mainly observed in the meninges but rarely inside the tumor mass. Conversely, the significant accumulation of EYFP^+^ microglia in the tumor as well as the subsequent reduction of this density at late disease stages were highlighted only by microscopic imaging. The microglial cells present in the healthy surrounding tissue collected for cytometry most likely attenuated and masked the changes of intratumoral densities.

Microscopic imaging can thus characterize the tumor microenvironment, particularly the representation and dynamic distribution of immune cells, as a regulator of malignancy. The specificity of our fluorescent markers in the early stages of the disease as well as characteristic morphological and kinematic features of the different populations of immune cells have allowed us to distinguish between resident microglia and neutrophils or monocyte-derived populations.

In our reporter mouse model EYFP^+^ cells were nearly all CD45^low^/CD11b^high^ in the sham control and in early phases of tumor growth. They were never observed in the blood stream. They were also positive for Iba1, had ramified morphology and were thus considered as brain microglial resident cells^8^. EYFP^+^ microglia represented a small fraction of the total microglia, likely in a weakly activated state. Indeed MHCII^+^ was weakly expressed at D21 and at D28 was preferentially encountered in the non-labeled population. In immunostained images, only 20% were EYFP^+^ Iba1^+^ MHCII^+^, characterizing activated microglia. Moreover, of the three typical classes of morphologies reported for microglial activation states^30^, we indeed found mainly star shaped microglia. However, microglial activation was boosted during tumor development as indicated by the progressive prevalence of the amoeboid phenotype despite the significant decline of microglial cell densities. Phagocytic events by EYFP^+^ microglia were equally observed at all stages. Our data did not support previous observations suggesting that microglia lose their capacity to express MHCII within the GBM microenvironment^31^.

By contrast, at D28, cytometric analysis identified 40% of the EYFP^+^ cells as peripheral CD45^high^/CD11b^low^/CD11c^+^/MHCII^+^ cells rather than microglia. This is consistent with the massive recruitment of the XCR1^+^ DCs usually reported in tumors^32^. MHCII immunostaining indicated that XCR1^+^ DCs were exclusively located in the meninges and superficial tumor areas at D21 then accumulated at the tumor margins by D28. Such meningeal and peritumoral localization is consistent with the proposed role of XCR1^+^ DCs that hyperactivate CD8^+^ T cells during tumor development^33^, while T cells accumulate in the meningeal and choroid plexus vessels, potential gateways for immune cells into the brain^34^. Interestingly, we also observed a stream of Iba1^−^/MHCII^low^/EYFP^+^ cells in the corpus callosum that targets the deepest areas of the tumor in the cortex. This would be compatible with an infiltration by immature peripheral DCs originating from the ventricles of the depth of the tumor and their differentiation into XCR1^+^/MHCII^+^ phenotype along their migration through the tumor toward the meninges. This differential distribution pattern in the CNS is reminiscent of data reported for the injured spinal cord describing different functional phenotypes for meningeal (inflammatory M1) and ventricular (anti-inflammatory M2)macrophages^35^. Therefore, our results strongly suggest that the combination of transgenic EYFP labeling and 3D intratumoral localization can serve the identification of immune cells in the brain at all stages of tumor development.

Similarly, EGFP was confirmed as a marker of cells of myeloid origin by our cytometry experiments. At early time points, neutrophils outnumbered monocytes/macrophages in brain cell suspensions but their density inside the tumor was in fact always at least five-fold lower than that of monocytes/macrophages as indicated by local dynamic intravital imaging and immunostaining data. The fastest moving cells observed in the tumor were most likely these few neutrophils that are recruited in a wide variety of murine tumor models^36^. Nevertheless, monocytes were the main population of circulating cells that accumulated inside the tumor when microglial cells density decreased. They represented the source of peripheral macrophages (P4/P5) and monocyte-derived DCs (P2/P3) inside the tumor according to the characterization of similar cell populations in the skin^15^. Being extremely plastic in their function and phenotype according to the microenvironment^37^,^38^,^39^, the long-lived monocytic cells are thus likely the key regulators of anti-tumor immune responses. In agreement, imaging showed that fluorescent cells acquired distinct morphologies and migratory properties depending on the time and location of the immune responses. This suggests that the analysis of cell morphologies and migration signatures based on the direction, speed, persistence and percentage of moving cells, could have broad implications for advancing our understanding of the inflammatory response.

It is likely that the tumor microenvironment progressively impeded the maturation of monocyte-derived cells, a possible cause for the fatal evolution of the tumor disease^40^,^41^. If brain monocytes can no longer exert direct elimination of tumor cells and cross-presentation of antigens to T-cells, tumor progression can be facilitated^42^.

We show here that co-expression of EYFP and EGFP in monocyte-derived cells could be used as a biomarker of this immune transition in order to stage disease progression. The highest incidence of co-expression occurred in the most mature subpopulations, namely P3 antigen presenting and P5 phagocytic monocyte-derived DCs. However, approximately 20% of the predominant less mature P2 monocyte-derived DC subpopulation also co-expressed EYFP and EGFP at D28.

It was noteworthy that the inflammatory environment induced by a GBM tumor in the CNS, like dermis inflammation in the ear^15^, favored the differentiation of CCR2^+^ monocytes into a CCR2^+^ DC P2 or P3 phenotype. This is in contrast with the differentiation into a macrophagic CCR2^−^ P4 or P5 phenotype encountered in the inflammatory microenvironment of a sterile liver injury^43^. A possible explanation could be that the antigen-free environment does not require an adaptative immune response, in contrast to tumors. Monocyte–derived DCs exhibit poorer T cell priming abilities than conventional DCs^15^, yet they accounted for 30% of the total DC population and therefore possibly impacted the adaptive antitumor immune response.

In the future, the targeted and specific ablation of XCR1^+^ DCs and monocyte-derived subpopulations *in vivo* should help to pinpoint their exact functions in brain immunity. This should aid in our understanding of the disease and actions of potential therapies on these processes. In support of the potential translational interest of our observations, we also showed that 50% of human GBM expressed LysM and that 58% expressed CD11c with spatial distributions in accord with observations made at different stages of tumor development in the mouse.

## Methods

### *In vivo* experiments

#### Animal care guidelines

All experimental procedures were performed in accordance with the French legislation and in compliance with the European Community Council Directive of November 24, 1986 (86/609/EEC) for the care and use of laboratory animals. The research on animals was authorized by the Direction Départementale des Services Vétérinaires des Bouches-du-Rhône (license D-13-055-21) and approved by the National Committee for Ethic in Animal Experimentation (Section N°14; project 87-04122012).

C57Bl6 mice (n = 97) were housed in cages with food and water *ad libitum* in a 12 h light/dark cycle at 22 ± 1 °C.

#### Cell culture and gene transfection

GL261 cells, a murine GBM cell line^44^ (National Cancer Institute, Charles River Labs), were transfected with a plasmid encoding for DsRed (pDsRed2-N1, Clontech, Mountain View, USA). Cells that stably express DsRed were selected using Geneticin (0.5 mg/ml, Gibco) and cultured as monolayers in RPMI1640 + GlutaMAX-1 (Gibco 61870) supplemented with 10% Fetal Calf Serum (Thermo Scientific) and Geneticin (0.5 mg/ml, Gibco). Cells were kept at 37 °C in a 5% CO2 atmosphere. Confluent cells were plated on Petri dishes coated with 1% soft agarose to induce spheroid formation as described in^24^.

#### Animal model

LysM-EGFP^21^, CD11c-EYFP^22^ and Thy1-CFP^45^ mice were used alone or crossed to obtain double transgenic mice.

The GBM animal model was realized as previously described^24^,^46^. Briefly, mice were anaesthetized by an intraperitoneal injection of a mixture of Xylazine/Ketamine (10 mg/kg and 100 mg/kg, respectively) and positioned on a stereotactic frame. A 3–4 mm diameter craniotomy was performed on the left parietal bone. The dura-mater was incised by a 31G needle and a 200–250 μm spheroid of GL261-DsRed murine GBM cells was injected into the cerebral cortex approximately 250 μm below the brain surface. A Sephadex hemi-bead with diameter fitting with the dura-mater opening was inserted in the injection wound and glued using histo-compatible acrylic glue (Cyanolit). A round glass coverslip (diameter: 5 mm) was then sealed on the adjacent bone and fixed to the skull by dental cement. Animals were left to recover for 15 days post-surgery before the first imaging session.

#### Experimental timetable

The experimental timetable is summarized in Suppl. Fig. 7. Mice were imaged every 3 to 4 days from day 15 until day 33 post-surgery. When animals showed a body weight loss of more than 15% of their original body weight, they were euthanized prior to this endpoint. For timelapse and cytometry experiments, animals were observed at day 21 and day 28.

#### Microscopy

Prior to each imaging session, mice were anaesthetized by an intraperitoneal injection of a mixture of Xylazine/Ketamine (10 mg/kg and 100 mg/kg, respectively), injected intravenously with 100 μl of a Cascade Blue conjugate dextran (70 kDa) solution (20 mg/ml in phosphate saline buffer (Sigma)) and positioned on a stereotactic frame allowing movements in the three-directions. Repositioning of the mice was realized using visual vascular landmarks to ensure that the same area is observed at each imaging session. The anesthesia is sufficient for a one hour observation. However, for timelapse experiments, the anesthesia was switched to a continuous inhalation of isoflurane (0.2 to 0.75% in humidified air) after the first hour of observation.

We used a Zeiss LSM 7MP two-photon microscope home-modified to allow animal positioning below the 20X water immersion objective (1.0 NA) and coupled to a femtosecond pulsed infrared tunable laser (Mai-Tai, Spectra Physics). After two-photon excitation, signals were epicollected and separated by dichroic mirrors and filters on 5 independent non-descanned detectors (collecting range: 390–485 nm, 500–520 nm, 520–549 nm, 555–605 nm and 605–678 nm)^29^. Images were acquired sequentially using an excitation wavelength tuned at 800 nm and then at 940 nm. Gains and offsets were identical for all the detectors, except for the red channels whose gain was reduced by 30% to compensate for the very strong expression of DsRed in tumor cells.

For CD11c-EYFP and LysM-EGFP cells recruitment experiments, images were typically acquired over a depth of 500 μm using 20 μm steps. Laser intensity was linearly increased with depth. Images were acquired as mosaics in order to cover the whole tumor surface with the healthy peritumoral tissue that surrounds it.

For time-lapse experiments, stacks of images were acquired from 20 to 200 μm below the dura-matter with a z-step of 3 μm. The acquisition was realized over 2 to 3 hours with time steps of 5 min. Ultra-fast timelapse experiments were also realized over a period of 10 min at a 0.1 Hz repetition rate.

#### Data analysis

For tumor growth measurements, the red channel (555–605 nm) from each mosaic z-stack was projected and the resulting area was measured as an index of the tumor size. Tumor size was plotted against time and an exponential curve was fitted. Fits were considered as valid if the determination coefficient (R^2^) was >0.5^46^. The mean cell density (number of cells/mm^2^) was calculated at each time points from the CD11c-EYFP and LysM-EGFP cells. Counts were obtained in 3 to 5 representative regions of interest inside the tumor selected at different depths from 0 to 250 μm below the dura-matter.

Timelapse z-stacks were processed using the open-source software Fiji and dedicated macros. Hyperstacks were first registered in 3D and cells of interest were manually tracked in 3D using the plugin MTrackJ^47^. Time-color coded images were generated using the temporal-color code plugin of Fiji. The resulting stack was then projected to obtain an image where immobile cells appear in white whereas mobile cells appear in color.

#### Cytometry

Animals were deeply anaesthetized by an intraperitoneal injection of a mixture of Xylazine/Ketamine, and were perfused by cardiac injection of 20 mL of PBS 1X. Brains were extracted and stored in ice-cold PBS. Olfactory bulbs and cerebellum were discarded and the cerebral hemisphere containing the tumor was excised. Cerebral tissues located below the corpus callosum including ventricles were removed with forceps. Then, tumor and surrounding healthy cerebral cortex including dura-mater were stored in a tube with DNAse I, Collagenase D (Roche Applied Science) and Collagenase V (Sigma). The dissociation was realized on a GentleMACS Octo Dissociator (Miltenyl Biotec), the suspension was then filtered while enzymatic reaction was blocked with EDTA. The cell suspension was centrifuged in an 80%/40% Percoll density gradient and rinsed before incubation with 2.4G2. Finally, the cell suspension was incubated 20 min with a mix of antibodies (see Suppl. Table 1) and labeled with Sytox Blue (Life technology). Acquisition was realized on a 5 lasers BD LSRFortessa^TM^ FACS equipment and data were extracted using the BD FACSDiva software . All acquisition are done in a standardized way using application settings. The gating strategy used to extract immune cells is described in Suppl. Fig. 3.

#### Patient characteristics and tumor samples

One hundred and twenty three patients (80 males and 43 females) with GBM (GBM, WHO grade IV) were included in this retrospective study. Patient age at the time of surgery ranged from 20 to 81 years (mean age at surgery: 59.2 years ± 14.3 years). Complete or subtotal surgical excision was performed in 72 cases, incomplete surgical excision (partial or biopsy) in 49 cases, excision quality was unknown in 2 cases. Tumor specimens were obtained according to a protocol approved by the local institutional review board and ethics committee and conducted according to national regulations. All the patients provided written informed consent. GBM samples provided from the AP-HM tumor bank (authorization AC-2013-1786 and 2014-A00585-42) were pooled on a tissue-microarray (TMA) for high throughput screening. The histology of the paraffin-embedded samples was confirmed by a pathologist (DFB).

#### Immunohistochemistry

For mice, brains were extracted and fixed overnight in 4% paraformaldehyde. Then, they were cryoprotected by a 24 h bath in 30% sucrose. 25 μm thick slices were realized on a cryotome and permeabilized in a 0.5% Triton solution. A one-hour blockage of the non-specific sites was realized (bovine serum albumin 2%, goat serum 2%, donkey serum 2%, Triton 0.1%) and primary antibodies were incubated overnight at 4 °C in PBS (rabbit anti-Iba1, 1/200, Wako (019-19741); rat anti-MHCII, 1/50, Ebiosciences (145321); rat anti-Ly6G, 1/50, Ebiosciences (145931)). Secondary antibodies (goat anti-rabbit conjugated to Dylight405, 1/100, Thermo (35551); donkey anti-rat conjugated to Cy5, 1/100, Jackson Immunoresearch (712-175-150) were incubated 1 h 30 at room temperature. Slices were then mounted with Vectashield. Slices were washed in PBS in between the different steps. Observations were performed on a Zeiss LSM780 confocal microscope in the spectral mode, using 405, 488, 543 and 633 nm excitation wavelength and spectral deconvolution processing of the images.

For human biopsies, after steam-heat-induced antigen retrieval, 5-μm sections of formalin-fixed paraffin-embedded samples were tested for the presence of LysM (EPR2994(2), 1/1000, rabbit IgG, GeneTex, Inc, Irvine, USA), CD11c (EP1347Y, 1/400, rabbit IgG, Abcam, Paris, France), Iba1 (1/1000, rabbit IgG, Wako Chemicals GmbH, Germany) and CD11b (EP1345Y, 1/100, rabbit IgG, Abcam). A Benchmark Ventana autostainer (Ventana Medical Systems SA, Illkirch, France) was used for detection, and slides were simultaneously immunostained to avoid inter-manipulation variability. Slides were then scanned (Nanozoomer 2.0-HT, Hamamatsu Photonics SARL France, Massy, France) and images processed in NDP.view2 software (Hamamatsu). Results of the stainings were determined from three areas of the tumor for each patient and then expressed as negative or positive.

### Statistical analysis

All data are expressed as mean ± SEM. Mann-Whitney U-test or one-way ANOVA were used to test differences in between time points. *p* < 0.05 was used as a criterion for *significance, while *p* < 0.001 was used as a criterion for ***significance. All statistical analyses were performed with Microsoft Excel and Graphpad Prism.

To analyze data on the patient cohort, analyses were conducted using the statistical package SPSS software v.17 (SPSS Inc, Chicago, USA). To compare patients’ groups according to immunoexpressed inflammation markers, we used Pearson’s chi-square test and Fisher’s exact test as appropriate. Progression free survival (PFS) and overall survival (OS) of the patients were calculated from the date of surgery until disease progression, death or last follow-up (censured data). Survival was estimated using the Kaplan–Meier method and curves were compared using the log-rank test. All the tests were two-sided and *p*-values of less than 0.05 were considered significant for each statistical analysis.

## Additional Information

**How to cite this article**: Ricard, C. *et al*. Phenotypic dynamics of microglial and monocyte-derived cells in glioblastoma-bearing mice. *Sci. Rep*. **6**, 26381; doi: 10.1038/srep26381 (2016).

## Supplementary Material

Supplementary Information

Supplementary Video S1

Supplementary Video S2

Supplementary Video S3
