# Supplementary material for: Phenotypic dynamics of microglial and monocyte-derived cells in glioblastoma-bearing mice
Source: Sci Rep. 2016 May 19;6:26381. doi: 10.1038/srep26381 (PMC4872227; doi:10.1038/srep26381)
Supplement: Supplementary Information [file srep26381-s1.pdf]

SUPPLEMENTARY FIGURES, TABLES AND VIDEOS

PHENOTYPIC DYNAMICS OF MICROGLIAL AND MONOCYTE-DERIVED CELLS IN GLIOBLASTOMA-BEARING MICE.

Clément Ricard[1,2,3,4], Aurélie Tchoghandjian[2,4], Hervé Luche[5], Pierre Grenot[5,], Dominique Figarella-Branger[2,4], Geneviève Rougon[# 1,3] , Marie Malissen[# 5,6] & Franck Debarbieux[#,* 1,3].

1. Institut des Neurosciences de la Timone, Marseille, Aix-Marseille Université and CNRS UMR7289, France.

2. Services d'Anatomie Pathologique-Neuropathologique et de Pharmacie, Assistance Publique – Hopitaux de Marseille, Marseille, France.

3. Centre Européen de Recherche en Imagerie Médicale, Aix-Marseille Université, Marseille, France.

4. Centre de Recherche en Oncobiologie et Oncopharmacologie, INSERM UMR911 and Aix-Marseille Université , Marseille, France.

5. Centre d'Immunophénomique, Aix-Marseille Université UM2, INSERM, US012, CNRS UMS3367, Marseille, France.

6. Centre d'Immunologie de Marseille-Luminy, Aix Marseille Université UM2, INSERM, U1104, CNRS UMR7280, Marseille, France.

[#] *equivalent participation*

* *corresponding author*

Figure S1

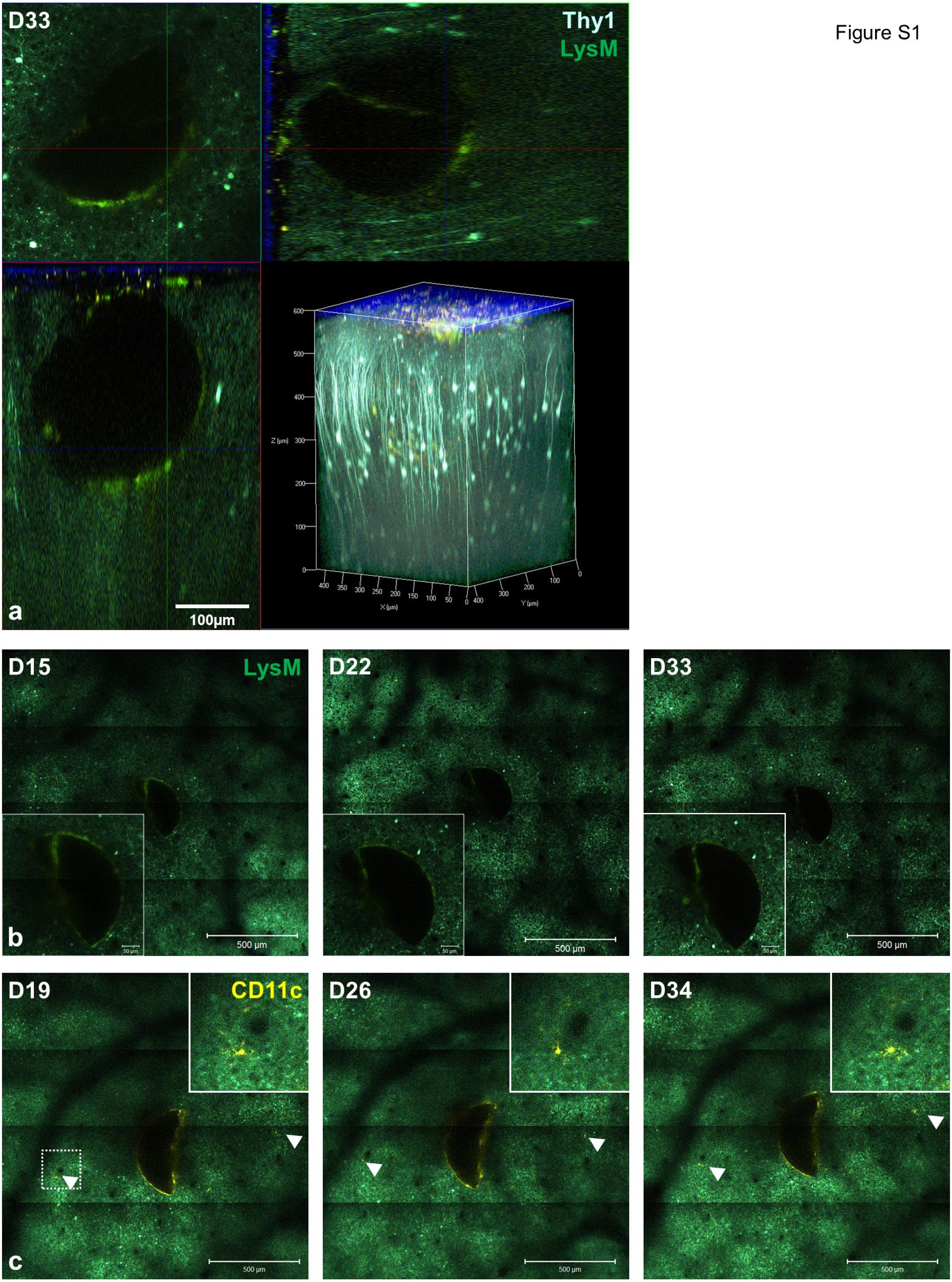
D33
Thy1
LysM
a
100µm
Z (µm)
X (µm)
Y (µm)
D15
LysM
D22
D33
b
500 µm
50 µm
D19
CD11c
D26
D34
c
500 µm

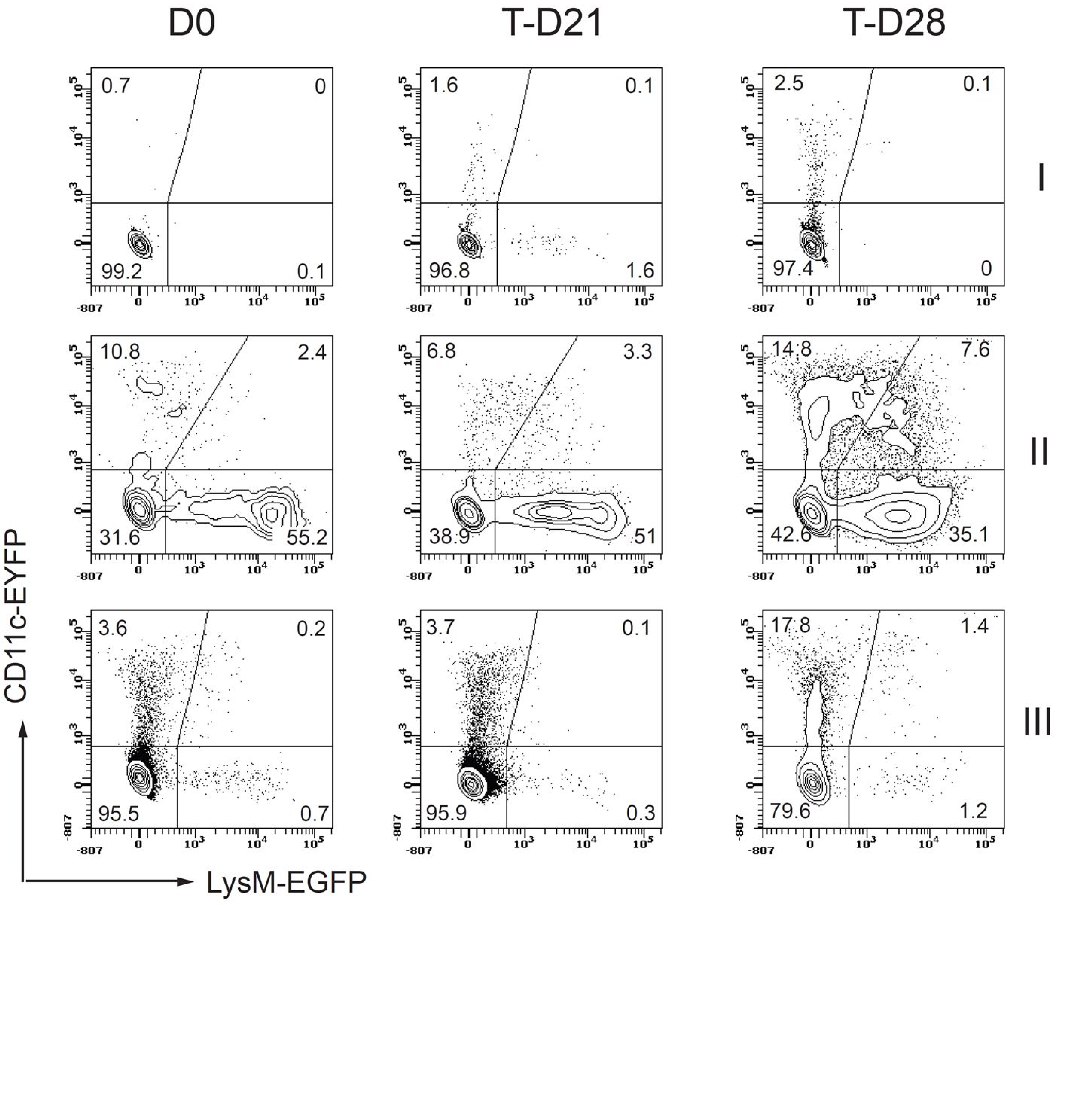

D0
T-D21
T-D28
I
II
III
CD11c-EYFP
LysM-EGFP
0.7
0
99.2
0.1
1.6
0.1
96.8
1.6
2.5
0.1
97.4
0
10.8
2.4
31.6
55.2
6.8
3.3
38.9
51
14.8
7.6
42.6
35.1
3.6
0.2
95.5
0.7
3.7
0.1
95.9
0.3
17.8
1.4
79.6
1.2

Figure S2

Figure S3

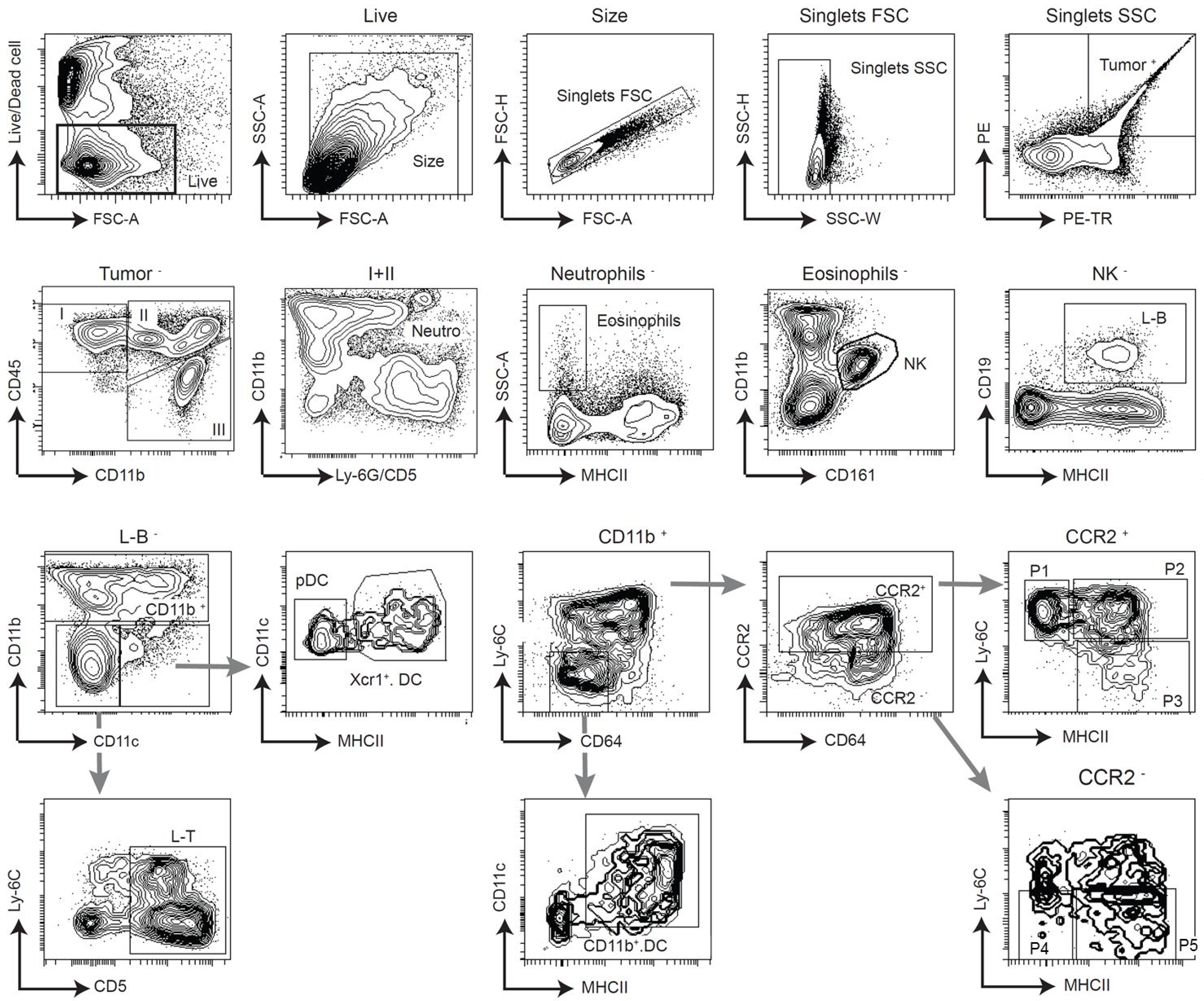

Live
Size
Singlets FSC
Singlets SSC
Live/Dead cell
FSC-A
Live
SSC-A
Size
FSC-H
SSC-H
SSC-W
PE
PE-TR
Tumor +
Tumor -
I+II
Neutrophils -
Eosinophils -
NK -
CD45
CD11b
I
II
III
Ly-6G/CD5
Neutro
SSC-A
MHCII
Eosinophils
CD161
NK
CD19
L-B
L-B -
CD11b +
CD11c
pDC
Xcr1+. DC
Ly-6C
CD64
CCR2
CCR2+
CCR2 -
CCR2 +
P1
P2
P3
CCR2 -
P4
P5
CD5
L-T
CD11b+.DC

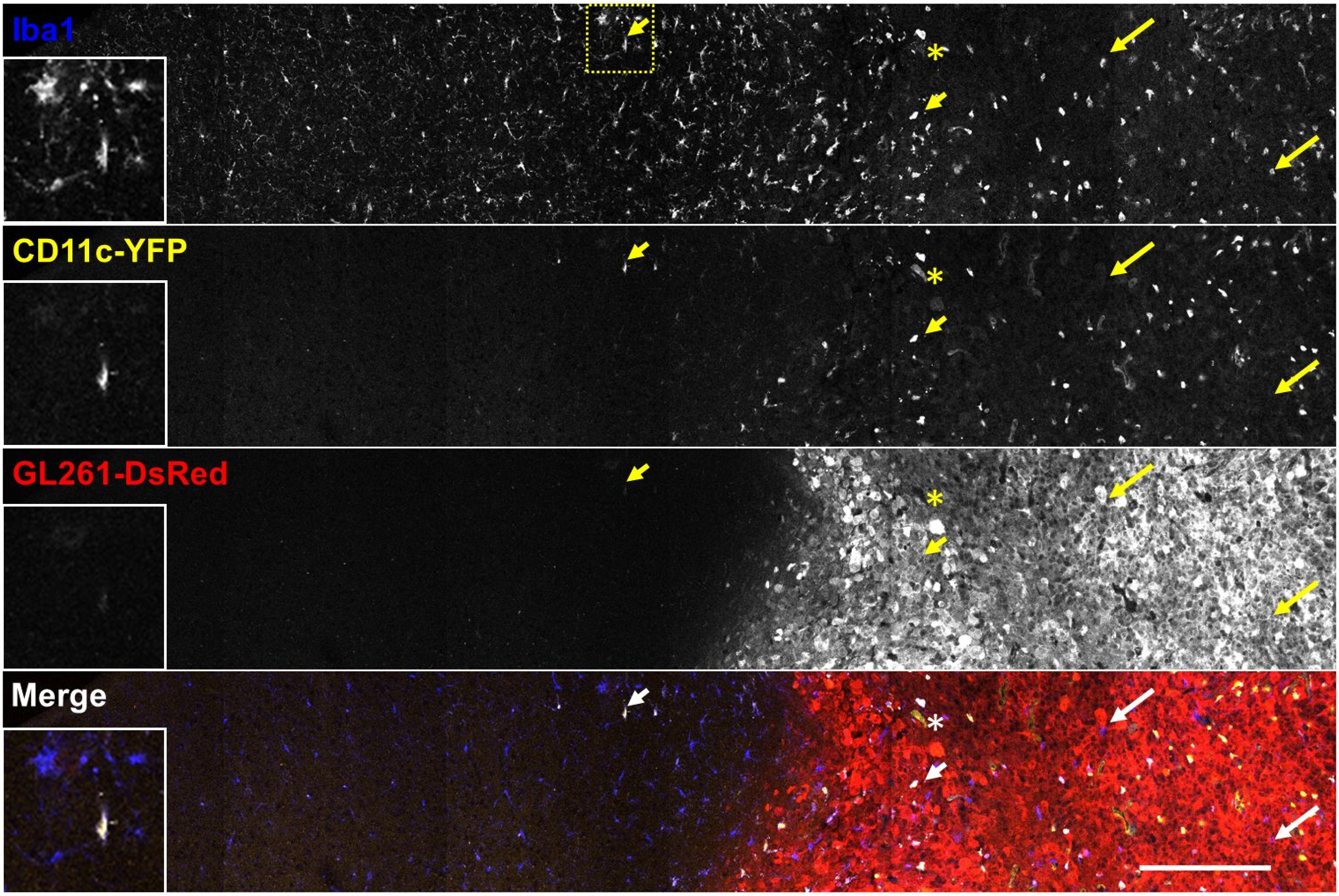

Figure S4

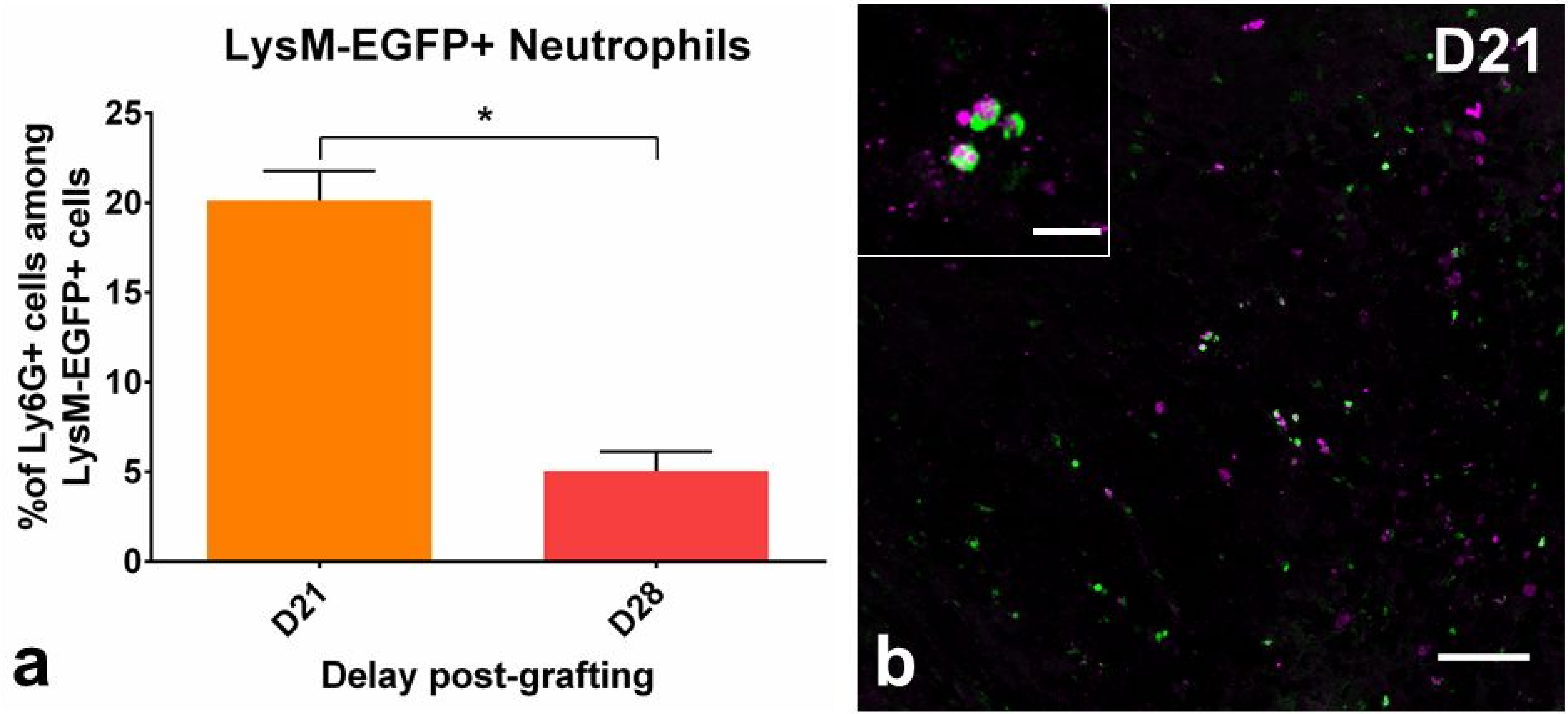
LysM-EGFP+ Neutrophils
%of Ly6G+ cells among LysM-EGFP+ cells
25
20
15
10
5
0
*
D21
D28
Delay post-grafting
a
D21
b

Figure S5

D21

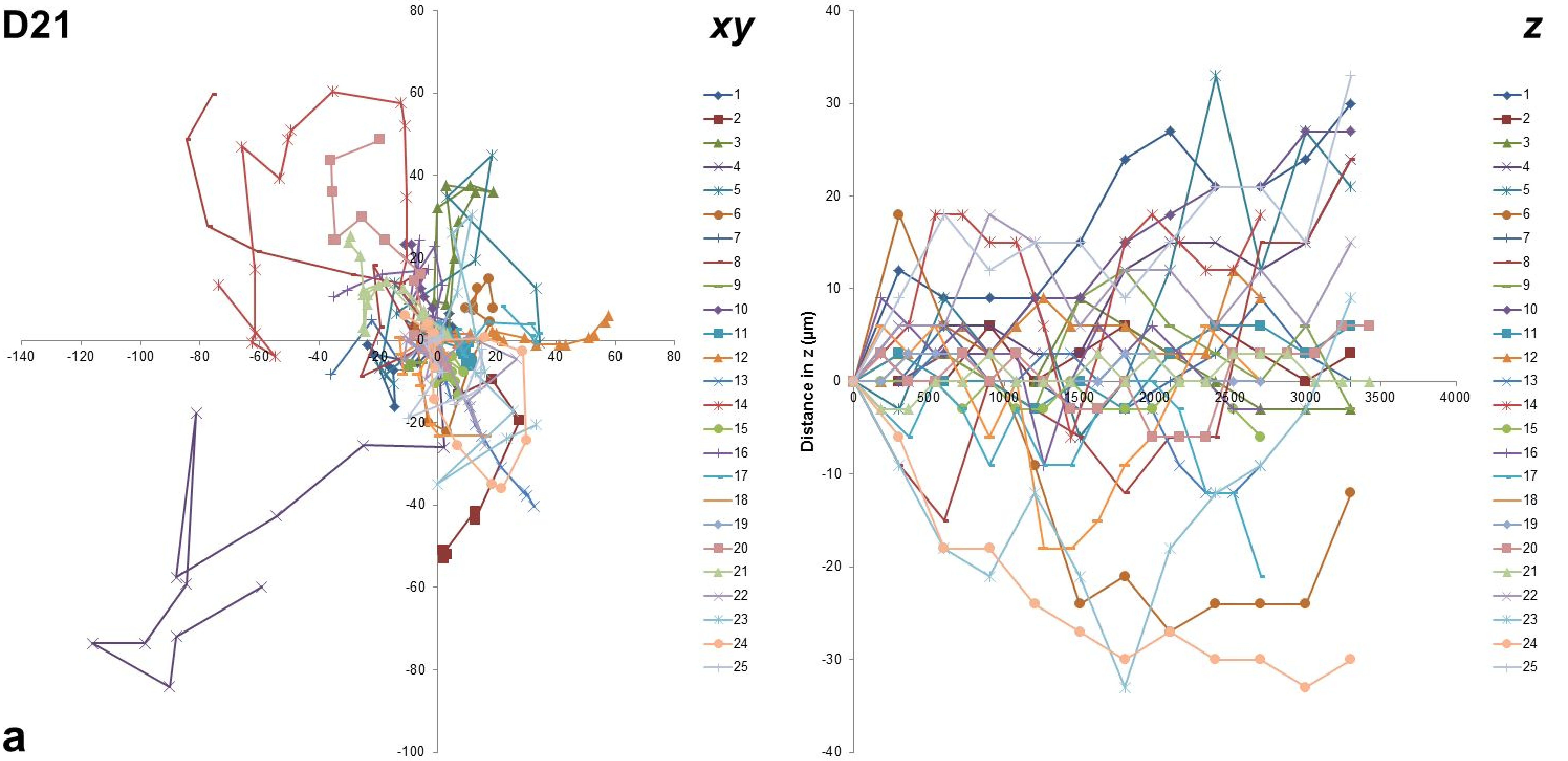

a

D28

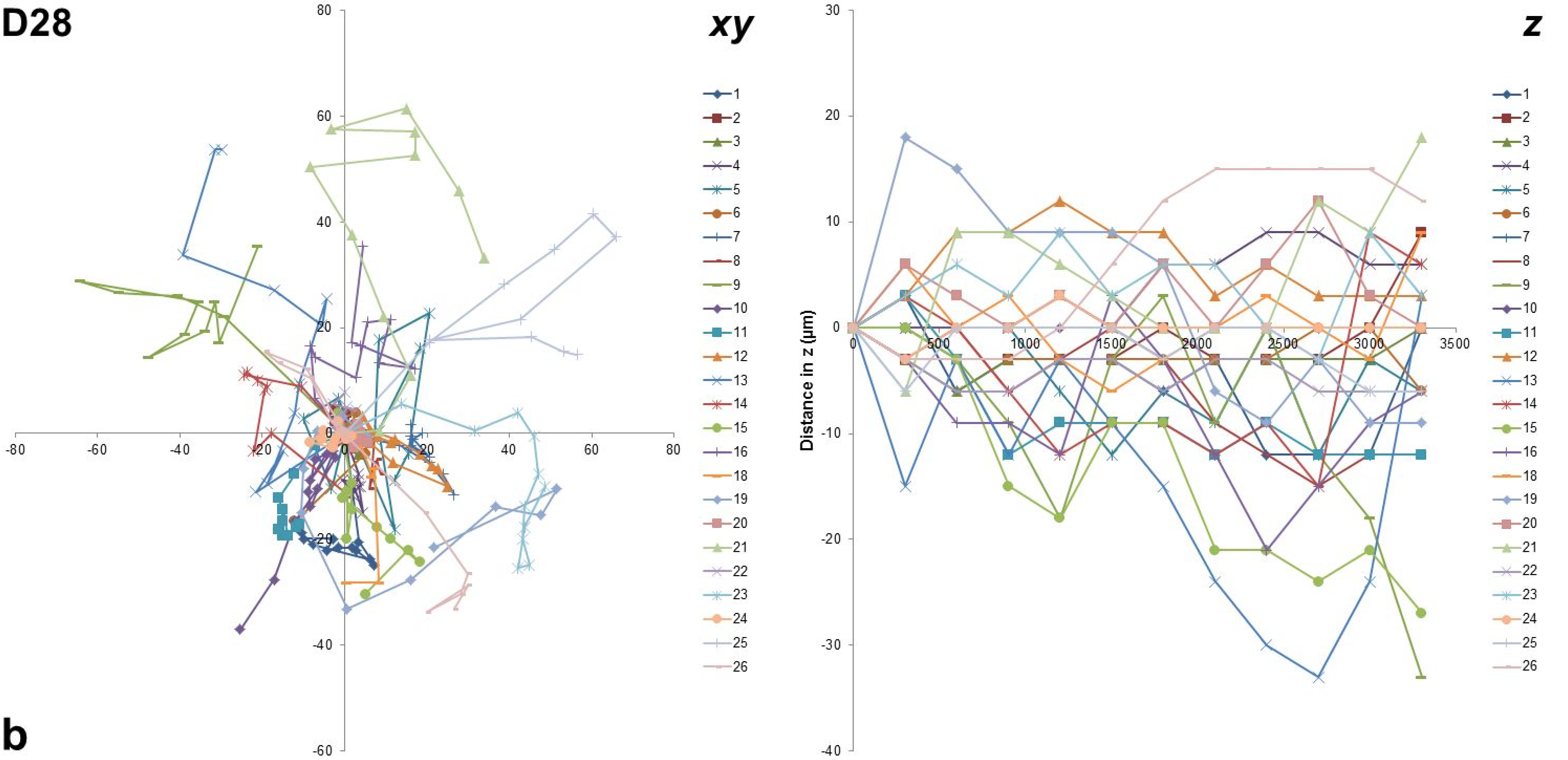

b

D28

LysM-EGFP Instant Speed

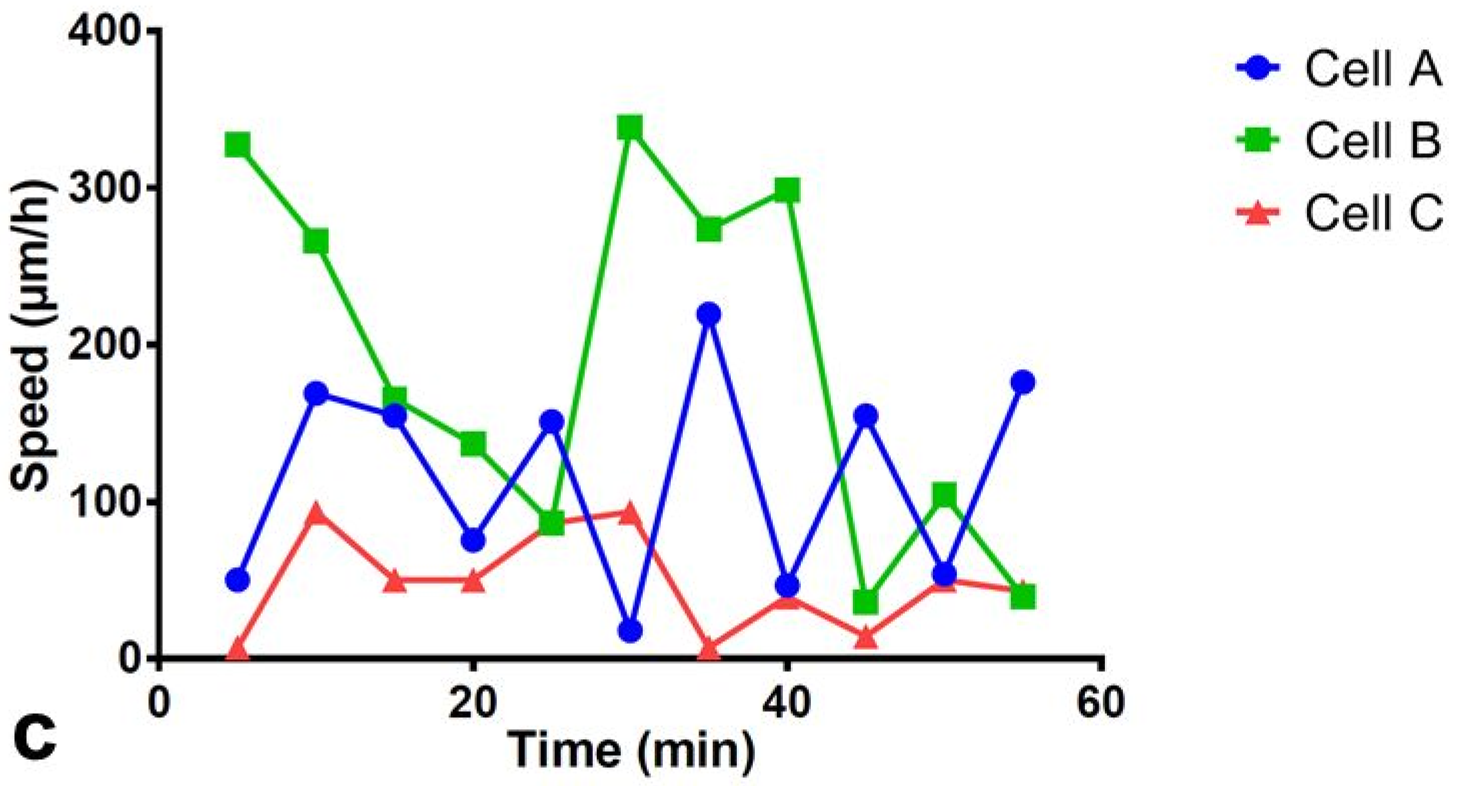

c

Figure S6

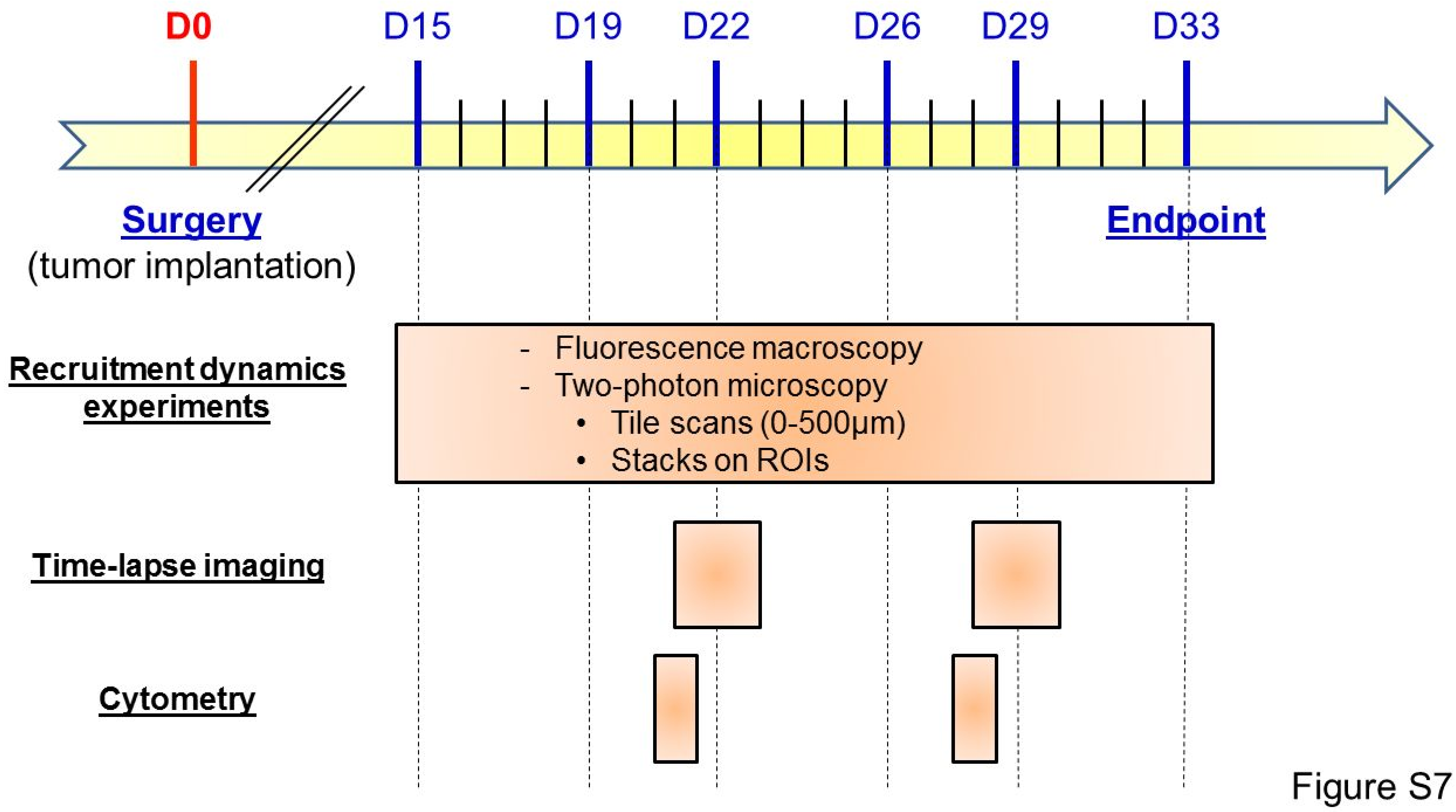
D0
D15
D19
D22
D26
D29
D33
Surgery
(tumor implantation)
Endpoint
Recruitment dynamics experiments
- Fluorescence macroscopy
- Two-photon microscopy
• Tile scans (0-500µm)
• Stacks on ROIs
Time-lapse imaging
Cytometry

Figure S7

Supplementary table 1 :

Molecule	Supplier	Clone
**Siglec H**	eBioscience	
**CD5**	BD Bioscience	53-7.3
**LY6C**	Biolegend/Ozyme	HK1.4
**CD45**	Biolegend/Ozyme	30F11
**CD161**	Biolegend/Ozyme	NK11
**CD11b**	BD Bioscience	M1/70
**CD11c**	Biolegend/Ozyme	N418
**LysM-GFP**		
**CD11c-EYFP**		
**CCR2**	R&D	475301
**Siglec F**	BD Bioscience	E50-2440
**Ds-Red2**		
**CD19**	eBioscience	1D3
**F4/80**	Biolegend/Ozyme	BM8
**CD64**	Biolegend/Ozyme	X54-5/7.1
**CD8a**	BD Bioscience	53-6.7
**LY6G**	BD Bioscience	1A8
**CMHCII**	Biolegend/Ozyme	M5/114.15.2
**Live/Dead**		

SUPPLEMENTARY FIGURES LEGENDS

Suppl. Fig.1: Intravital two-photon imaging of CD11c-EYFP and LysM-EGFP cell dynamics in sham-operated animals.

(**a**) Orthogonal reconstruction and 3D-rendering after intravital two-photon imaging of a Thy1-CFP (neurons)/LysM-EGFP (myeloid cells) mouse at D33 post-surgery. Scale-bar: 100µm. (**b**) Intravital two-photon imaging of a Thy1-CFP/LysM-EGFP mouse at D15, D22 and D33 post-surgery. Some LysM-EGFP cells lay on the Sephadex hemi-bead but do not accumulate in the surrounding tissue. Scale-bar: 500µm. Insets: zoom on the Sephadex hemi-bead. n=6 mice. Scale-bar: 50µm. (**c**) Intravital two-photon imaging of a Thy1-CFP (neurons) /CD11c-EYFP (microglia, XCR1^{+} DCs) mouse at D19, D26 and D34 post-surgery. Some CD11c-EYFP^{+} cells lay on the Sephadex hemi-bead but are rare in the surrounding tissues (arrows) and not attracted by the Sephadex hemi-bead over time. Scale-bar: 500µm. Insets: zoom on a CD11c-EYFP^{+} cell that is stable over time (n=6 mice). Scale-bar: 50µm. Blue: second-harmonic generation (dura-mater); cyan: neurons; green: LysM-EGFP^{+} cells; yellow: CD11c-EYFP^{+} cells; dark area: Sephadex hemi-bead.

Suppl. Fig.2: Expression of EGFP and EYFP in cell populations I, II and III. Cells corresponding to populations I, II and III were extracted from the brain before tumor grafting (D0) or 21 (T-D21) or 28 days (T-D28) after tumor grafting and analyzed for the expression of the LysM-EGFP and CD11b-EYFP fluorescent reporters. The percentage of EGFP^{-}EYFP^{-}, EGFP^{-}EYFP^{+}, EGFP^{+}EYFP^{+} and EGFP^{+}EYFP^{-} cells are indicated in each plot.

Suppl. Fig.3: Gating strategy used for analyzing the cells contained within the brain.

Cell suspensions were prepared by enzymatic digestion of the brain. Among Sytox Blue-negative cells (gate live cells), single cells (gate singlets) were first selected from which tumor cells (Tumor^{+}) were excluded on the basis of their fluorescence (DsRed expressing tumor). The remaining CD45$^{+/low}$ leukocytes Tumor^{-} cells were further analyzed. The microglia (III), neutrophils, eosinophils, NK cells and B cells were successively excluded from the CD45$^{+/low}$ Tumor^{-} cells. The remaining cells were then analyzed. CD45$^{+/low}$ Tumor^{-} cells deprived of microglia (III), neutrophils, eosinophils, NK

cells and B cells were separated on the basis of CD11c and CD11b into three populations. CD11c⁻CD11b⁻ cells correspond mainly to CD5⁺ T cells, CD11c⁺CD11b⁻ cells comprise pDC and Xcr1⁺DC, whereas CD11b⁺ cells comprise Ly-6C⁻ CD64⁻ CD11b⁺DC and monocytes, moDCs and macrophages (denoted as P1 to P5 as in[15]).

Suppl. Fig.4: CD11c-YFP⁺ cells characterization in brain parenchyma. Iba1 immunostaining (blue) of a CD11c-EYFP (yellow) mouse cortex bearing a GL261 Ds-Red glioma (red). Arrows: Iba1⁺ cells that do not colocalize with CD11c-YFP; arrowheads: Iba1⁺ cells that colocalize with CD11c-YFP; asterisk: CD11c-YFP cell that does not colocalize with Iba1 staining. n=2 mice and 4 ROI per animal were used for the quantification. Scale-bar: 100µm.

Suppl. Fig.5: Neutrophil quantification inside the tumor. (**a**) Percentage of Ly6G⁺ cells among LysM-EGFP⁺ cells at D21 and D28 by quantitative immunohistochemistry. *: $p<0.05$, Mann-Whitney test. (**b**) Representative immunohistochemistry image taken at D21. Green: LysM-EGFP; magenta: Ly6G. Scale-bar: 100µm (inset: 20µm).

Suppl. Fig.6: Amoeboid LysM-EGFP cells have no preferential direction in the tumor core. (**a-b**) xy (left) and z (right) tracking of LysM-EGFP amoeboid cells in the tumor core at (**a**) Day 21 (D21, n=25 cells) and (**b**) Day 28 (D28, n=26 cells). The origin of the coordinates (0,0,0) was systematically set as the origin of the trajectories at t=0 and tracked for 55min. Graph units: x,y,z: µm; t: seconds. (c) Evolutions of instantaneous speed for 3 individual LysM-EGFP⁺ cells showing irregular kinetics with phases of arrest (speed < 20micron/h).

Suppl. Fig.7: Experimental timetable.

Suppl. Table.1: Antibodies used for cytometry experiments.

SUPPLEMENTARY MOVIES LEGENDS

Suppl. Movie 1: Intravital two-photon timelapse imaging over a ten minutes observation period at D21. In the center, note the CD11c-EYFP microglial cells that wrap around tumor cells and transiently interact with a LysM-EGFP⁺ cell. Large

deformations of a LysM-EGFP$^+$ cell are visible. Time resolution: 10s. Red: Tumor, Green: LysM-EGFP$^+$ cells, Yellow: CD11c-EYFP$^+$ cells. Scale-bar: 20µm.

Suppl. Movie 2: Intravital two-photon timelapse imaging over a five minutes observation period at D28. Note the motile CD11c-EYFP cell (most probably a DC, arrow) whose soma moves during the acquisition period and that expands cytoplasmic protrusions over glioma cells. Deformations of a LysM-EGFP cell (arrowhead) are also highlighted. Time resolution: 1sec. Red: Tumor, Green: LysM-EGFP$^+$ cells, Yellow: CD11c-EYFP$^+$ cells. Scale-bar: 20µm.

Suppl. Movie 3: Intravital two-photon time-lapse imaging over a three-hour observation period at D28. Note the CD11c-EYFP$^+$ cells wrapping around tumor cells that acts as a focal spot for interactions with LysM-EGFP$^+$ cells over a few hours. Time resolution: 5min. Red: Tumor, Green: LysM-EGFP$^+$ cells, Yellow: CD11c-EYFP$^+$ cells. Scale-bar: 20µm.
